# Immunotherapy of SARS based on combinations of neutralizing human monoclonal antibodies

**DOI:** 10.2217/fvl.09.78

**Published:** 2010-03-01

**Authors:** Lanying Du, Shibo Jiang

**Affiliations:** Lindsley F Kimball Research Institute, New York Blood Center, New York, NY 10065, USA. ldu@nybloodcenter.org; Lindsley F Kimball Research Institute, New York Blood Center, New York, NY 10065, USA. Tel.: +1 212 570 3058; Fax: +1 212 570 3099; sjiang@nybloodcenter.org

## Abstract

**Evaluation of: Coughlin MM, Babcook J, Prabhakar BS: Human monoclonal antibodies to SARS-coronavirus inhibit infection by different mechanisms. *Virology* 394(1), 39–46 (2009).** This work discusses passive immunotherapy based on neutralizing human monoclonal antibodies (mAbs) with different mechanisms of action. The authors have demonstrated that combining such mAbs, which target distinct epitopes, may greatly increase inhibition of virus infection and suppress the generation of neutralization escape mutants. The inhibition of human mAbs to SARS-coronavirus (CoV) may also act through different mechanisms of action, depending on their target epitopes or regions. Therefore, this approach could provide fast and effective prophylaxis and treatment of SARS-CoV infection during a SARS outbreak. Specifically, Coughlin *et al.* have indicated that most of the tested anti-S1 mAbs recognized epitopes within the receptor-binding domain and blocked virus attachment to its cellular receptor. These findings could provide a further step in understanding the mechanism of these mAbs in the prevention of SARS-CoV infection, as well as an insight into the design and development of novel therapeutic treatments.

Immunization is an effective method against the re-emergence of SARS. Recently developed SARS vaccines have shown effectiveness in animal models and some clinical trials [1–3]. However, owing to the very low incidence of SARS infection since 2004, it could be very costly to vaccinate a large susceptible population. A more reasonable, rapid and cost-effective alternative under these circumstances could be the implementation of passive immunotherapy, in which human neutralizing monoclonal antibodies (mAbs) would play a key role in prevention and treatment. Neutralizing mAbs have demonstrated efficacy as a prophylaxis against a variety of viral infections [4]. Currently, some neutralizing mAbs to SARS have been tested in animal models and were proven to be effective in protecting against SARS-coronavirus (CoV) infection [5,6]. This has made it possible to use passive transfer of neutralizing mAbs to prevent the quick spread of SARS-CoV in the case of regional outbreak. However, a key task involves understanding their underlying mechanisms of action.

Using XenoMouse™, a human immunoglobulin transgenic mouse, Coughlin *et al.* previously produced a series of neutralizing human mAbs against the S protein of SARS-CoV, in which they bound epitopes within or upstream (residues 12–261) of the receptor-binding domain (RBD) [7]. In the present study, the authors have focused on understanding the antiviral mechanisms of these mAbs. To accomplish this, they first developed a receptor binding inhibition assay with Vero E6 cells naturally expressing the receptor, angiotensin-converting enzyme (ACE)2, to detect the inhibition of mAbs to viral attachment. Each of the 19 previously identified S1-specific mAbs was preincubated with a purified protein expressing S1 of SARS-CoV fused with Fc of human IgG (S12–510-Fc). This was then added to Vero E6 cells and detection of protein binding in the presence of mAbs to the target cell surface via flow cytometry was performed. Their results demonstrated that 18 of these anti-S1 mAbs, designated as group 1, recognized seven distinct epitopes within the RBD and that all were capable of efficiently inhibiting S12–510-Fc protein binding. These findings suggest that the mechanism of these group 1 mAbs might involve the inhibition of SARS-CoV infection by blocking viral attachment to the cellular receptor of target cells [8]. Thus, it appears that they possess a receptor-blocking mechanism similar to mAbs S227.14, S230.15 and 80R, as previously reported by Rockx *et al.* and Sui *et al.*, respectively [9,10]. mAbs S227.14 and S230.15 recognize partially overlapping epitopes coinciding with RBD, while 80R binds to a conformationally sensitive fragment (residues 324–503) overlapping the RBD [10,11].

Since one of the 19 anti-S1 mAbs failed to block the binding of the S12–510-Fc protein to the cellular receptor, its antiviral activity might, therefore, be a consequence of other mechanisms. This mAb was designated as 4D4, belonging to group 2 mAbs binding upstream of RBD. Thus, the authors generated a pseudovirus system containing HIV backbone and expressing the SARS-CoV S protein (HIV/S), and used it to examine the ability of mAb 4D4 to inhibit viral entry. The pseudovirus was bound to 293T/T17 cells transiently transfected with human receptor ACE2 at 4°C for 1 h, allowing viral attachment (binding), but not entry. Then the cells were treated with increasing concentrations of mAb 4D4 and incubated at 37°C until luciferase activity was detected. This way, Coughlin *et al.* demonstrated that mAb 4D4 inhibited a postbinding step in viral entry and that such postbinding inhibition was significantly greater than that indicated by direct preincubation of pseudovirus with mAb before adding to the target cells [8]. Therefore, this mAb might prevent the conformational change necessary for S protein cleavage by cathepsin.

In this study, the authors further tested the efficacy of combining mAbs to inhibit SARS-CoV entry. In doing so, they combined mAb 4D4 with different RBD-specific mAbs from group 1, including 3C7, 5D3, 5E4 and 3H12, and tested the neutralization via the HIV/S pseudotyped virus assay. Their results revealed that all these mAb combinations demonstrated a significant increase in inhibition. A similar increase of protection was detected by combining mAbs 4D4 and 3C7 to neutralize live SARS-CoV (Urbani) infection in Vero E6 cells [8]. These results suggest that combining antibodies containing distinct epitopes and neutralizing virus with different mechanisms may result in a greater inhibition of virus infection.

Escape mutants can be generated in the presence of mAbs. In the study by Coughlin *et al.*, the authors tested the ability of 11 mAbs to yield escape mutants by preincubation of individual mAbs with SARS-CoV (Urbani) and incubating the mixture in Vero E6 cells with nine passages of virus supernatant. They found that escape mutants emerged in the presence of nine of the 11 mAbs at different passages of virus culture, suggesting that these mAbs possessed different abilities in selecting escape mutants when used individually. However, these escape mutant viruses could be effectively neutralized by a mixture of mAbs consisting of 3H12, 4D4 and 3C7, indicating that a cocktail of mAbs may be effective in the suppression of escape mutants. These results are consistent with previous reports by ter Meulen *et al.*, who demonstrated that the mixture of two noncompeting human mAbs, CR3014 and CR3022, which recognize different epitopes in RBD, may neutralize SARS-CoV infection in a synergistic fashion, potentially controlling immune escape and extending the breadth of protection [12].

## Conclusion & future perspective

Passive immunotherapy driven by neutralizing human mAbs could be an effective candidate for prophylaxis and treatment of SARS-CoV infection. Neutralizing antibody responses against SARS-CoV S protein could be broadly elicited after virus infection, demonstrating long-lasting immunity in the sera of most recovered SARS patients [13]. Some human mAbs to SARS-CoV have been evaluated in animal models and demonstrated efficacy. Human mAb 80R, for example, may largely reduce viral replication when given as a prophylaxis to mice at doses therapeutically achievable in humans [11]. According to Roberts *et al.*, therapy with neutralizing human mAb 201 to SARS-CoV reduced disease severity and viral burden in Golden Syrian hamsters [5]. This fact suggests that neutralizing mAbs may be used as an important emergent prophylaxis to help rapidly clear the virus, thus providing immediate protection of the human population in the event of a SARS outbreak.

As previously mentioned, the mechanism of mAbs as antiviral agents may vary, depending on their targeting epitopes or regions. Most of the mAbs targeting epitopes within RBD, as reported by Coughlin *et al.* and others [8,14], interfere with virus–receptor interactions by blocking attachment of the virus to the target cells, thus providing protection from SARS-CoV replication. Some other mAbs, as tested by Coughlin *et al.* in this study, target the S1 domain upstream of RBD by inhibiting viral entry through a postbinding event. Since human mAbs inhibit SARS-CoV infection by different mechanisms, combining mAbs with various mechanisms of inhibition and targeting multiple antigenic epitopes may induce additive or synergistic effect, largely reducing the possibility that neutralization escape mutants can be generated. Studies by Rockx *et al.* have indicated that a cocktail of three mAbs (S109.8, S227.14 and S230.15), which target distinct epitopes, completely protected aged mice against weight loss and virus replication in the lungs following lethal challenge of a live SARS-CoV icHC/SZ/61/03, minimizing the likely generation of escape mutants [10]. The enhancement of protective effect by combinational mAbs recognizing distinct neutralizing epitopes was further confirmed in the current study [8]. Overall, understanding of the mechanisms of action underlying the antiviral activity of these mAbs will be instrumental in the design and development of novel therapeutic treatments against SARS-CoV infection.

In conclusion, immunotherapies with humanized protective antibodies have been shown to have prophylactic and therapeutic potential for SARS-CoV infections in animal models, thus providing an important basis for clinical trials of these mAbs in human populations. The use of SARS-CoV-specific human mAbs as a prophylaxis may provide a safe, feasible and efficacious way to prevent virus infection, thus serving as an alternative and significant intervention to vaccines. However, caution should be taken when using neutralizing human mAbs as an immunoprophylaxis strategy, in which a genotyping surveillance of SARS-CoV-like genotypes in known animal reservoirs might be required [11]. It should be noted that passive transfer of antibodies may be less efficient in protecting immunosenescent populations than young populations. It is expected that even more effective neutralizing mAbs, targeting multiple epitopes of SARS-CoV containing different mechanisms, could be developed and tested for the prevention of SARS infection and the treatment of SARS patients.

**Executive summary**

**Objectives of this study**

- To understand the mechanism of human monoclonal antibodies (mAbs) that recognize epitopes within and upstream of the receptor-binding domain in the inhibition of SARS-coronavirus (CoV) replication. To detect the efficacy of antibody combinations in suppressing virus escape mutants and in neutralizing virus infection.

**Methods**

- A receptor-binding inhibition assay with Vero E6 cells naturally expressing receptor angiotensin-converting enzyme (ACE)2 to detect the inhibition of mAbs to the binding between S1 protein and its receptor, ACE2.
- A pseudotyped SARS-CoV neutralization assay based on HIV backbone and SARS-CoV S protein to detect the postbinding inhibition of mAbs and to test the efficacy of combining mAbs to inhibit SARS-CoV entry.
- A neutralizing assay based on live SARS-CoV (Urbani) to detect protection of mAb combination against SARS-CoV (Urbani) and to detect mAb mixture in suppressing escape mutant viruses in virus-infected Vero E6 cells.

**Conclusion**

- A mixture containing mAbs recognizing distinct regions and targeting multiple steps of viral entry is more effective than individual mAb being used alone in neutralizing SARS-CoV infection and suppressing mutant virus generation, thus serving as a potential passive immunotherapy.

## References

[1] Martin JE, Louder MK, Holman LA *et al.*: A SARS DNA vaccine induces neutralizing antibody and cellular immune responses in healthy adults in a Phase I clinical trial. *Vaccine* 26(50) 6338–6343 (2008). 18824060 10.1016/j.vaccine.2008.09.026 PMC2612543

[2] Lin JT, Zhang JS, Su N *et al.*: Safety and immunogenicity from a Phase I trial of inactivated severe acute respiratory syndrome coronavirus vaccine. *Antivir. Ther.* 12(7) 1107–1113 (2007). 18018769

[3] Ishii K, Hasegawa H, Nagata N *et al.*: Neutralizing antibody against severe acute respiratory syndrome (SARS)-coronavirus spike is highly effective for the protection of mice in the murine SARS model. *Microbiol. Immunol.* 53(2) 75–82 (2009). 19291090 10.1111/j.1348-0421.2008.00097.x PMC7168451

[4] Marasco WA, Sui J: The growth and potential of human antiviral monoclonal antibody therapeutics. *Nat. Biotechnol.* 25(12) 1421–1434 (2007). 18066039 10.1038/nbt1363 PMC7097443

[5] Roberts A, Thomas WD, Guarner J *et al.*: Therapy with a severe acute respiratory syndrome-associated coronavirus-neutralizing human monoclonal antibody reduces disease severity and viral burden in golden Syrian hamsters. *J. Infect. Dis.* 193(5) 685–692 (2006). 16453264 10.1086/500143 PMC7109703

[6] ter Meulen J, Bakker AB, van den Brink EN *et al.*: Human monoclonal antibody as prophylaxis for SARS coronavirus infection in ferrets. *Lancet* 363(9427) 2139–2141 (2004). 15220038 10.1016/S0140-6736(04)16506-9 PMC7112500

[7] Coughlin MM, Lou G, Martinez O *et al.*: Generation and characterization of human monoclonal neutralizing antibodies with distinct binding and sequence features against SARS coronavirus using XenoMouse. *Virology* 361(1) 93–102 (2007). 17161858 10.1016/j.virol.2006.09.029 PMC7103293

[8] Coughlin MM, Babcook J, Prabhakar BS: Human monoclonal antibodies to SARS-coronavirus inhibit infection by different mechanisms. *Virology* 394(1) 39–46 (2009). 19748648 10.1016/j.virol.2009.07.028 PMC7111986

[9] Sui J, Li W, Murakami A *et al.*: Potent neutralization of severe acute respiratory syndrome (SARS) coronavirus by a human mAb to S1 protein that blocks receptor association. *Proc. Natl Acad. Sci. USA* 101(8) 2536–2541 (2004). 14983044 10.1073/pnas.0307140101 PMC356985

[10] Rockx B, Corti D, Donaldson E *et al.*: Structural basis for potent cross-neutralizing human monoclonal antibody protection against lethal human and zoonotic severe acute respiratory syndrome coronavirus challenge. *J. Virol.* 82(7) 3220–3235 (2008). 18199635 10.1128/JVI.02377-07 PMC2268459

[11] Sui J, Li W, Roberts A *et al.*: Evaluation of human monoclonal antibody 80R for immunoprophylaxis of severe acute respiratory syndrome by an animal study, epitope mapping, and analysis of spike variants. *J. Virol.* 79(10) 5900–5906 (2005). 15857975 10.1128/JVI.79.10.5900-5906.2005 PMC1091676

[12] ter Meulen J, van den Brink EN, Poon LL *et al.*: Human monoclonal antibody combination against SARS coronavirus: synergy and coverage of escape mutants. *PLoS Med.* 3(7) e237 (2006). 16796401 10.1371/journal.pmed.0030237 PMC1483912

[13] Temperton NJ, Chan PK, Simmons G *et al.*: Longitudinally profiling neutralizing antibody response to SARS coronavirus with pseudotypes. *Emerg. Infect. Dis.* 11(3) 411–416 (2005). 15757556 10.3201/eid1103.040906 PMC3298259

[14] Greenough TC, Babcock GJ, Roberts A *et al.*: Development and characterization of a severe acute respiratory syndrome-associated coronavirus-neutralizing human monoclonal antibody that provides effective immunoprophylaxis in mice. *J. Infect. Dis.* 191(4) 507–514 (2005). 15655773 10.1086/427242 PMC7110081

